# Biofilm Produced In Vitro by *Piscirickettsia salmonis* Generates Differential Cytotoxicity Levels and Expression Patterns of Immune Genes in the Atlantic Salmon Cell Line SHK-1

**DOI:** 10.3390/microorganisms8101609

**Published:** 2020-10-20

**Authors:** Natacha Santibañez, Matías Vega, Tatiana Pérez, Alejandro Yáñez, Roxana González-Stegmaier, Jaime Figueroa, Ricardo Enríquez, Cristian Oliver, Alex Romero

**Affiliations:** 1 Laboratorio de Inmunología y Estrés de Organismos Acuáticos, Instituto de Patología Animal, Facultad de Ciencias Veterinarias, Universidad Austral de Chile, Campus Isla Teja, Valdivia 5090000, Chile; natacha.santi@gmail.com (N.S.); matias.vega.n@gmail.com (M.V.); tatybioq@gmail.com (T.P.); rgstegmaier@gmail.com (R.G.-S.); renrique@uach.cl (R.E.); 2 Interdisciplinary Center for Aquaculture Research (INCAR), Concepción 4070386, Chile; ayanez@uach.cl (A.Y.); jefigueroa@uach.cl (J.F.); 3 Facultad de Ciencias, Universidad Austral de Chile, Valdivia 5090000, Chile; 4 Instituto de Bioquímica y Microbiología, Facultad de Ciencias, Universidad Austral de Chile, Valdivia 5090000, Chile

**Keywords:** *Piscirickettsia salmonis*, SRS, biofilm, gene expression, bacterial virulence

## Abstract

*Piscirickettsia salmonis* is the causative agent of Piscirickettsiosis, an infectious disease with a high economic impact on the Chilean salmonid aquaculture industry. This bacterium produces biofilm as a potential resistance and persistence strategy against stressful environmental stimuli. However, the in vitro culture conditions that modulate biofilm formation as well as the effect of sessile bacteria on virulence and immune gene expression in host cells have not been described for *P. salmonis*. Therefore, this study aimed to analyze the biofilm formation by *P. salmonis* isolates under several NaCl and iron concentrations and to evaluate the virulence of planktonic and sessile bacteria, together with the immune gene expression induced by these bacterial conditions in an Atlantic salmon macrophage cell line. Our results showed that NaCl and Fe significantly increased biofilm production in the LF-89 type strain and EM-90-like isolates. Additionally, the planktonic EM-90 isolate and sessile LF-89 generated the highest virulence levels, associated with differential expression of *il-1β*, *il-8*, *nf-κb*, and *iκb*-α genes in SHK-1 cells. These results suggest that there is no single virulence pattern or gene expression profile induced by the planktonic or sessile condition of *P. salmonis*, which are dependent on each strain and bacterial condition used.

## 1. Introduction

Chilean fish farming has gone through a tremendous expansion over the last decades including the culture of three main salmonids species such as Atlantic salmon (*Salmo salar*), Coho salmon (*Oncorhynchus kisutch*), and rainbow trout (*O. mykiss*). Unfortunately, increased fish farming has been accompanied by the development of novel infectious-contagious diseases caused mainly by viral, parasitic, and bacterial pathogens. Piscirickettsiosis is the most prevalent bacterial disease for salmonids in Chilean aquaculture [1]. This disease is caused by *Piscirickettsia salmonis*, a γ-proteobacteria classified into two different genogroups associated with the LF-89^T^ (ATCC VR-1361) type strain and the Chilean isolate EM-90 [2,3]. *P. salmonis* possess the type IV secretion system [4,5], which secretes effector proteins during in vivo infection [6]. Likewise, *P. salmonis* secretes outer membrane vesicles (OMVs) [7], which transport several virulence proteins and toxins to the extracellular environment and within the host cell, and are involved in bacterial virulence [8].

Biofilm is a highly complex, well-organized three-dimensional structure and cooperating community of microorganisms, regulated by nutrient availability and several environmental factors such as osmolarity, pH, temperature, oxygen content, and iron [9,10,11,12]. Living in biofilms is a natural state of occurring bacteria [13], allowing complex interactions between cells and with the biofilm matrix: this grants emergent properties at the community level that are not apparent at the level of individual free-living cells. Thus, the matrix can also act as a “protective shield” as a survival and persistence strategy under stressful conditions [14]. This makes mechanical removal of bacteria organized in biofilms very difficult, demonstrating greater resistance to disinfectants and antimicrobials [15]. Isiaku et al. [16] and Thuptimdang et al. [17] reported that biofilms play an important role in bacterial pathogenicity, especially in chronic infections where the physical and spatial arrangement of these structures impede the access of bactericidal drugs; restricting their effects to the surface, making them extraordinarily resistant to phagocytosis, and increasing the difficulty of eradicating the biofilm from their hosts [18,19,20].

Marshall et al. [21] described that *P. salmonis* cultured in nutritional restricted media generated exopolysaccharides aggregates, which are similar to typical biofilm structures. This suggests that this pathogen may produce biofilms as a way to survive and persist in marine surroundings or simply as an independent defense feature against hostile environmental conditions. Similarly, several fish pathogens such as *Streptococcus phocae* [22], *Vibrio ordalii* [23], *Flavobacterium columnare* [24], and *F. psychrophilum* [25] can attach to different materials commonly found in fish farms and form biofilms, causing recurrent exposure to pathogens and, consequently, asymptomatic carriers [26,27,28]. Thus, bacterial adhesion and growth through biofilm production could be considered as a major virulence factor involved in the pathogenicity of *P. salmonis* [29]. *P. salmonis* express the chemotaxis operon cheYZA [30] and several genes involved in biofilm biosynthesis [3]. Interestingly, Levipán et al. [29] reported that two isolates of *P. salmonis* formed biofilms in seawater and AUSTRAL-SRS broth. This study also described the cytotoxicity generated by the biofilm of these isolates in SHK-1 cells. However, no published studies have evaluated the host immune response induced by *P. salmonis* biofilms. Thus, this study aimed to determine and analyze the biofilm formation of different *P. salmonis* isolates under several culture conditions and to evaluate the virulence of planktonic and sessile bacteria and the immune gene expression induced by these bacterial conditions in *Salmo salar* head kidney (SHK-1) cells. 

## 2. Materials and Methods

### 2.1. Bacterial Strains and Culture Conditions

*P. salmonis* LF-89^T^ (ATCC VR-1361) type strain and the Chilean isolates LF-89-like 1, EM-90-like 1, and EM-90-like 2 were routinely grown as a bacterial lawn on BBL™ Trypticase Soy Agar (Becton, Dickinson and Company, Sparks, MD, USA) plates supplemented with NaCl 15 g/L, fetal bovine serum (FBS) 5%, l-cysteine 0.1%, d-glucose 0.5%, and FeCl_3_ 0.120 mM at 18 °C for five days. After that, *P. salmonis* were carefully scraped from bacterial lawn and suspended onto 5 mL of Tryptic Soy Broth (TSB) (Merck, Darmstadt, Germany) supplemented with NaCl 3 g/L, FBS 2.5%, l-cysteine 0.05%, and FeCl_3_ 0.01 g/L and grown at 18 °C and 100 rpm for two days [31] prior to the biofilm formation assays. The identified strains were confirmed by PCR assays [30] and 16S rRNA sequencing [32]. 

### 2.2. Quantitative Assessment of P. salmonis Biofilm Formation in Restrictive Liquid Media

To test different culture conditions for the biofilm formation assays, a basal medium was established using the Marine Broth (MB, BD Difco, Franklin Lakes, NJ, USA) formulation as a reference, which was modified to obtain the following variations of NaCl (7.1, 24.6, and 32.1 g/L) and ferric citrate (0.01, 0.08, and 0.16 mM). To evaluate the *P. salmonis* biofilm formation, the bacteria were cultured in 96-well flat-bottom culture plates (TrueLine, Nippon Genetics, Tokyo, Japan) using 180 µL of media containing different NaCl and ferric citrate concentrations, which were then inoculated with 20 µL of bacterial inoculum (McFarland 0.5 standard, 1.5 × 10^8^/mL) and incubated at 18 °C without agitation for up to eight days. Non-inoculated media (200 µL) were used as negative controls. Biofilm formation was quantified after 0, 2, 4, and 8 days of culture by the crystal violet (CV) method [33,34] as follows: the culture medium was carefully removed from each well containing the adhered biofilm, which was subsequently washed with phosphate-buffered saline PBS 1× (200 µL) for 5 min to eliminate any non-adhered biofilm or live bacteria. Then, plates were incubated at 37 °C to fix the adhered biofilm, followed by incubation for 10 min with crystal violet (200 µL at 3 g/mL). The CV solution was then discarded, and the wells were washed with PBS 1× for 5 min by repeated pipetting to remove dye excess and dried upside-down at room temperature for 30 min [35]. Finally, images of the CV stained biofilm obtained for each culture condition were registered using an inverted microscope (100×) coupled to an Idu Labcam Microscopy Adapter. Biofilm formation was determined by reading the absorbance of solubilized CV at 595 nm using a Microplate Reader Synergy™ 2 (BioTek Instruments, Winooski, VT, USA).

To calculate the biofilm production (1), an OD cut-value (ODc) was established as proposed by Stepanovic et al. (2007) [34]. ODc was defined as three standard deviations (SD) above the mean OD of the negative control:

ODc = ODxn + 3 × SD,(1)

where ODxn is the average OD of the negative control and SD is the standard deviation of the negative control. Thus, the OD value of a tested strain was expressed as the average OD value of the strain or condition reduced by the ODc value. The ODc value was calculated for each microtiter plate separately. If a negative value was obtained, it was presented as zero, while any positive value indicated biofilm production.

### 2.3. Planktonic and Sessile P. salmonis Infection in SHK-1 Cells

To evaluate the effect of planktonic and sessile *P. salmonis* on cellular lysis, an in vitro infection assay was performed in SHK-1 cells. For infection, cells (passage between 61 to 63) were cultured without antibiotics in L-15 medium supplemented with 10% FBS at 20 °C in 75 cm^2^ flasks (Costar, Fisher Scientific, Ottawa, ON, Canada). Cells were seeded into 24-well plates at a concentration of 5 × 10^5^ cell per well for 24 h at 20 °C and infected with planktonic and sessile *P. salmonis* at multiplicity of infection (MOI) 10 (5 × 10^6^ bacteria) of LF-89 and isolate EM-90-like 2 strains. The bacterial inoculum was prepared by growing *P. salmonis* in MB medium (32.1 gr/L) at 18 °C for four days in six-well plates (sessile) or in 50 mL Falcon tubes (planktonic) under moderate agitation (100 rpm). Thus, planktonic and sessile bacteria were collected (100 μL) after four days of culture and counted using a LIVE/DEAD BacLight^TM^ Bacterial Viability Kit (Invitrogen, Molecular Probes, Eugene, OR, USA), following the manufacturer’s recommendations. For bacterial quantification, stained bacteria were placed on a Neubauer counting chamber and observed using a fluorescence microscope (Optika, B-1000 series) [36]. Bacteria with yellow-green and orange-red emissions were considered as living and dead (or inactive) bacteria, respectively. Cells were incubated for a further 17 days at 20 °C and non-infected cells were used as negative controls. Finally, cytotoxicity was evaluated as described by Oliver et al. [37] and RNA was isolated to evaluate immune gene expression.

### 2.4. Determination of Cytotoxicity by Lactate Dehydrogenase Assay

The cell damage induced by *P. salmonis* in SHK-1 cells was determined by evaluating the liberation of cytosolic enzyme lactate dehydrogenase (LDH) in the cell supernatant using a commercial Cytotoxicity LDH Detection Kit (Takara, Bio Inc., Otsu, Japan). LDH release levels were analyzed in 50 μL of cell-free supernatant from each cultured condition. Additionally, the supernatant of cells lysed with L-15 medium containing 1% Triton X-100 was used as a total lysis control (maximum LDH liberation) and the supernatant of uninfected cells was used as control cells. Optical density was measured at 490 nm and the result was obtained using the Equation (2):

% of cytotoxicity = [OD_490_ of (treated cells − control cells)]/[OD_490_ of (total lysis control cells − control cells)] × 100 (2)

### 2.5. RNA Purification and Gene Expression Analysis

Gene expression analysis was carried out through a kinetic study in cells infected with planktonic and sessile *P. salmonis*, as described above. The infected cells were collected at 2, 4, 6, 12, and 24 h, and total RNA extraction and cDNA synthesis were performed as described by Romero et al. (2012) [38]. LPS 5 μg/mL (Sigma-Aldrich, St. Louis, MO, USA) was used as a positive control for each time point. RNA was purified using the EZNA Total RNA (Omega Bio-Tek, Norcross, GA, USA) including the in-column DNAse I digestion step and the purified RNA was stored at −80 °C. Reverse transcription was performed using the M-MLV RT Kit (Promega) and the cDNA was stored at −20 °C. Gene expression was analyzed by qPCR using the Brilliant II SYBR^®^ Green qPCR Master Mix (Agilent Technologies, Santa Clara, CA, USA), and each reaction was performed using 250 nM of primers (Table 1) and 2.5 uL of 1:2 diluted cDNA as a template, in a QuantStudio 3 thermocycler (Applied Biosystems, Waltham, MA, USA). The fold change of gene expression was calculated according to the 2^−∆∆Ct^ method [39] using the expression of the elongation factor-1α (ef-1α) as a normalizer, and the expression of all markers in the control condition as a calibrator. The average and standard deviation of the fold change of gene expression were graphed and analyzed with the Tukey’s multiple comparison test (*p* < 0.05).

### 2.6. Statistical Analyses

Statistical analyses were carried out using GraphPad Prism^®^ 5 (GraphPad Software, Inc., La Jolla, CA, USA). The qPCR data were represented as fold change in the gene expression of cells treated with sessile or planktonic bacteria, compared to untreated control cells (*n* = 4). The data differences between the two culture conditions (sessile or planktonic) were analyzed using Tukey’s multiple comparison test (*p* < 0.05). The data are depicted as mean ± SD. Cell viability data are presented as means ± SD of four biological replicates and the data differences were calculated using the same statistical analysis.

## 3. Results

### 3.1. P. salmonis Biofilm Formation

**Figure 1:**
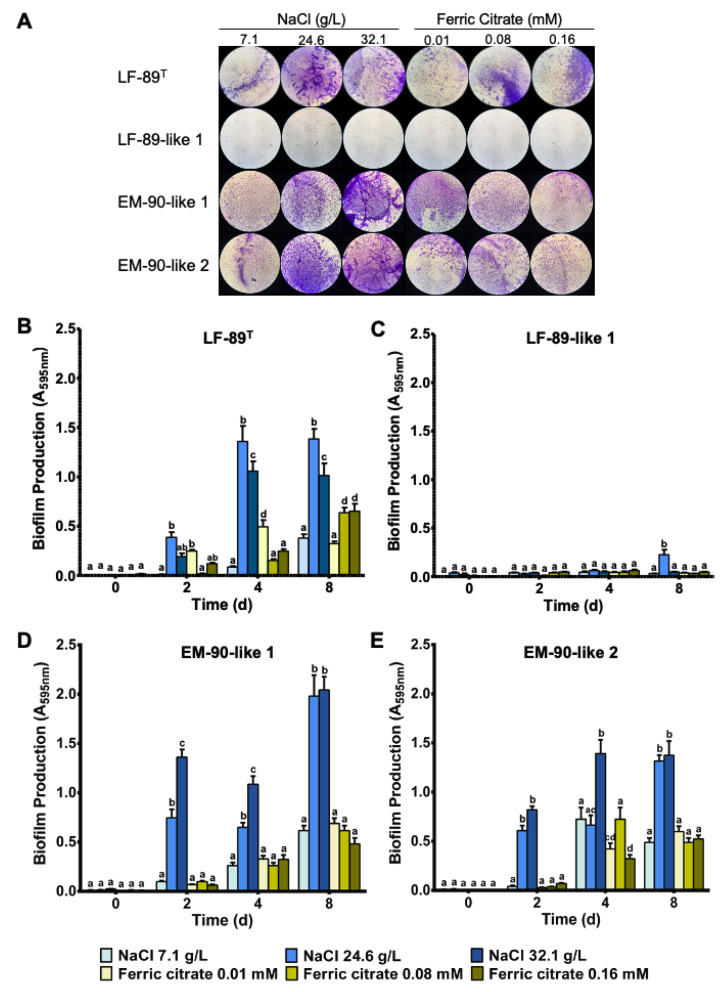
*P. salmonis* biofilm formation cultured under different NaCl and ferric citrate concentrations. (**A**) Representative CV staining of biofilm produced by *P. salmonis* LF-89 type strain and isolates LF-89-like 1, EM-90-like 1, and EM-like 2 at eight days (100× magnification). Biofilm quantification for *P. salmonis* LF-89 (**B**) and isolates LF-89-like 1 (**C**), EM-90-like 1 (**D**), and EM-90-like 2 (**E**) grown for 0, 2, 4, and 8 days. Values are presented as the mean ± SEM (*n* = 5). A two-way ANOVA followed by a Tukey HDS was performed to define the differences between media variation. Different letters indicate significant differences (*p* < 0.05).

To evaluate the effects of iron and salinity on *P. salmonis* biofilm formation, bacterial growth assays with increasing concentrations of ferric citrate or NaCl in MB medium were performed (Figure 1A). In the case of salinity, exposure of *P. salmonis* to high NaCl concentrations (26.4 g/L and 32.1 g/L) significantly increased biofilm formation in the LF-89 type strain (Figure 1B) and EM-90-like 1 and 2 (Figure 1D,E) at all evaluated culture times. Regarding iron concentrations, ferric citrate at 0.01, 0.08, and 0.16 mM also increased biofilm production in the LF-89 type strain (Figure 1B) and EM-90-like 1 and 2 (Figure 1D,E). However, these increases were lower compared to the biofilm formation obtained at a higher concentration of NaCl after four and eight days of culture. It is important to mention that the biofilm production for the Chilean isolate LF-89-like 1 showed the lowest biofilm formation values under all the conditions analyzed in this study (Figure 1A,C). However, the growth of this strain was similar to the other *P. salmonis* strains used.

### 3.2. Planktonic and Sessile P. salmonis Infection in SHK-1 Cells 

**Figure 2:**
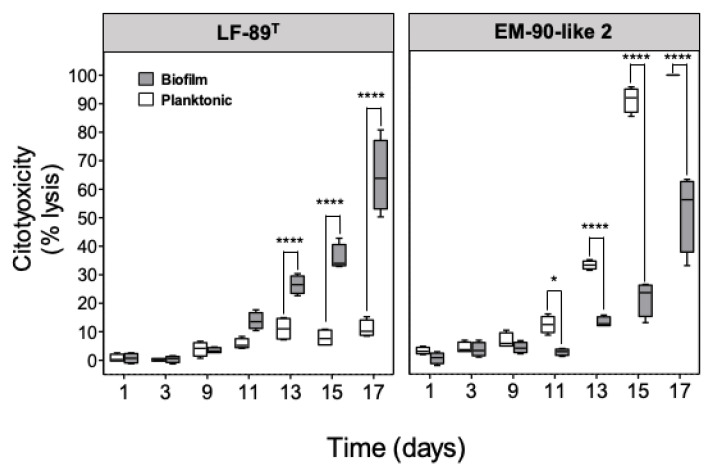
Cytotoxicity assay in SHK-1 cells infected with planktonic and sessile *P. salmonis*. SHK-1 cells were infected with planktonic or sessile *P. salmonis* (MOI = 10). Results are expressed as cytotoxicity percentage means ± standard error of each sample in triplicate. Asterisks indicate significant differences (* *p* < 0.05 and **** *p* < 0.001).

To evaluate the in vitro virulence generated by planktonic or sessile bacteria, a kinetic infection assay was performed using SHK-1 cells infected with the *P. salmonis* LF-89 type strain and EM-90-like 2 in both conditions. The viability of bacterial cells was evaluated by flow cytometry (Figure S1). The results showed independent cytotoxicity profiles for the strains in both conditions (Figure 2). Thus, although the cytotoxic effect of bacteria on cells tended to increase with time after infection with both bacterial strains (except for LF-89 planktonic), it is interesting to note that the effect of planktonic and sessile bacterial conditions was the opposite in the analyzed strains. Specifically, the sessile condition of LF-89 generated a sustained cytopathic effect, with increased cytotoxicity that reached about 60% after 17 days p.i compared to its planktonic condition, which caused only 10% of cellular lysis for the same time analyzed (Figure 2). In contrast, the sessile condition of the EM-90-like 2 strain generated only 20% cytotoxicity while the planktonic condition caused the highest effect in cellular lysis, which reached about 90% at day 15 post-infection (p.i).

### 3.3. Immune Gene Expression Analysis

**Figure 3:**
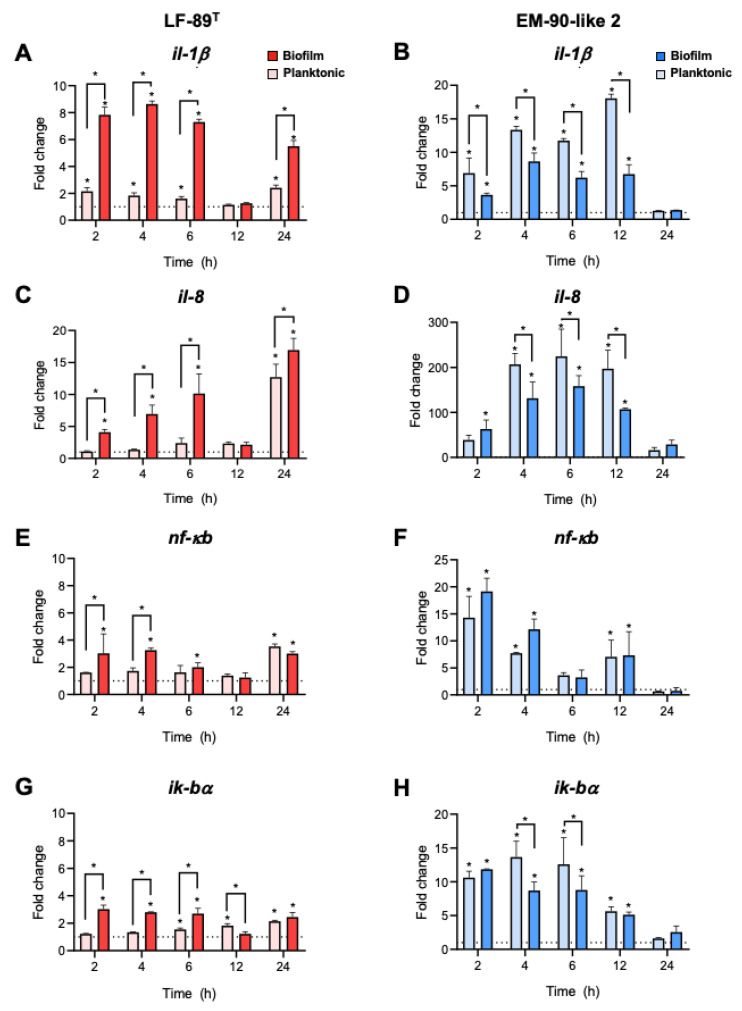
Analysis of the immune gene expression induced by planktonic and sessile *P. salmonis*. SHK-1 cells were infected with *P. salmonis* LF-89^T^ or EM-90-like 2 strain in planktonic or sessile conditions for 2, 4, 6, 12, and 24 h, and the mRNA levels of *il-1β* (**A**,**B**), *il-8* (**C**,**D**), *nf-κb* (**E**,**F**), and *iκb-α* (**G**,**H**) were analyzed by qRT-PCR. The expression of *ef-1α* was used as a normalizer. The graph depicts the average ± standard deviation of the gene expression fold change. Control cells were used as a calibrator (dotted line). Asterisks above the bars indicate statistically significant differences evaluated using Tukey’s multiple comparison test (*p* < 0.05).

To evaluate the effect of planktonic and sessile conditions on immune gene expression, an infection assay using two bacterial conditions of two different *P. salmonis* strains on SHK-1 cells was performed. The immune gene expression profile revealed differential modulation of these markers depending on the strain analyzed as well as on the sessile/planktonic bacterial condition (Figure 3). Cells infected with the sessile LF-89 type strain showed higher *il-1β* expression levels than its planktonic counterpart at 2, 4, and 6 h p.i. (Figure 3A). However, we observed the opposite effect in cells infected with the EM-90-like 2 strain at 2, 4, 6, and 12 h (Figure 3B). A strong upregulation of *il-8* was observed only in cells infected with the EM-90-like 2 strain, and this effect was higher when planktonic bacteria were used (Figure 3D). EM-90-like 2 strain presented increased expression of *nf-κb* at 2, 4, and 12 h (Figure 3F). Similarly, a strain-dependent upregulation was also observed for *iκb-α* gene expression. However, in this case, sessile bacteria generated a lower upregulation (Figure 3H). 

## 4. Discussion

One of the main objectives of this study was to determine the ability of *P. salmonis* to produce biofilm under different NaCl and ferric citrate concentrations in culture conditions. We found that the LF-89 type strain and the EM-90 type isolates generated biofilms in the modified MB culture medium, predefined as a medium that induces biofilm production in this pathogen [21,35]. An iron source was previously proposed as a necessary element for *P. salmonis* growth in vitro [40,41]. It is for this reason that lower concentrations than those described as optimal were chosen in our study, in order to induce an adverse environment that could trigger a bacterial defense response. However, the three different concentrations of ferric citrate used did generate minor differences in the biofilm production for the strains evaluated. The planktonic LF-89 type strain can grow normally at concentrations between 0.15 to 1 mM, while concentrations below 0.01 mM only decrease its growth by 25% [42]. Taking into account that biofilm formation is considered a mechanism of resistance to stressors [10], the iron concentrations used were possibly not low enough to induce strong biofilm formation in the bacteria in response to suboptimal concentrations. Likewise, we must consider the amount of iron present in both the yeast extract and meat peptone (MB components) at 55.3 ug/g and 0.004%, respectively, as an extra source for the bacteria. Additionally, the bacterium cultured in vitro lacks the pressure of the host, which, when faced with a bacterial infection, release an entire iron picking machinery as a way to decrease the accessibility of the bacteria to this metabolite [43].

The induced biofilm production in vitro by *P. salmonis* was initially described using MB medium [21,35], which contains a high salt concentration. In this context, our results revealed that NaCl variations showed greater differences in terms of biofilm formation for the bacteria. Thus, high NaCl concentrations (26.6 and 32.1 g/L), similar to those found in estuarine and marine environments, induced the highest biofilm production in the LF-89 strain and the EM-90 type isolates of *P. salmonis*. Lannan and Fryer [44] described the bacteria’s ability to survive in seawater for long periods of time with free-living bacteria that were found after 40 days in seawater [45]. The influence of salinity on biofilm production has been widely described on a variety of bacteria [46,47,48]. Although there is no predefined concentration that modulates the highest levels of biofilm production in general for bacteria, different studies have suggested an association between the optimal NaCl concentration existing in the natural environment of each microorganism and the concentration that increased the biofilm production used for in vitro assays. This is the case of *Flavobacterium columnare*, a freshwater bacterium responsible for columnaris disease: they produce greater amounts of biofilm in media with NaCl 5 g/L [24]. In the marine bacteria *Vibrio fischeri* and *Vibrio parahaemolyticus*, NaCl concentrations of 24.8 g/L and 20 g/L, respectively, induced the highest biofilm production [49,50]. Interestingly, the salt concentrations where these bacteria produced higher biofilm levels were similar to those concentrations in which the *P. salmonis* strains evaluated in our study produced higher biofilm levels. In this way, the in vitro results observed in this study could be extrapolated as an adaptive survival and persistence mechanism involving the development of extracellular aggregates by the bacteria, when submitted to adverse marine conditions.

From the results presented above, the next step to follow was to determine if the planktonic and sessile condition of *P. salmonis* had differential cytopathic effects in SHK-1 cells. Indeed, the infected cells showed differential damage (Figure 2), which can be influenced by both the planktonic or sessile condition as well as the bacterial strains used. This result is in agreement with what was described by Levipán et al. [29], who reported different cytotoxicity degrees between *P. salmonis* LF-89 and EM-90 isolates in their sessile and planktonic state. Most of the studies aiming to characterize *P. salmonis* virulence, pathogenicity, antimicrobial resistance, and induction of the immune responses are routinely conducted using planktonic bacteria or colonies suspended from plaque growth, inoculating SHK-1, ASK [51], and RTS11 [52] fish cell lines. SHK-1 cells are derived from leukocytes that have some of the properties of a macrophages [53], which are activated by several pathogen associated molecular patterns (PAMPs) such as lipopolysaccharides (LPS) present in bacterial surface and which are recognized by TOLL-like receptors (TLRs) present in these cells. In this way, they are capable of secreting proinflammatory cytokines. Likewise, the SHK-1 cells are able to phagocytose several fish pathogens such as *Aeromonas salmonicida* [53], ISA virus [54], and infectious pancreatic necrosis virus (IPNV) [55], among others, for the subsequent pathogen destruction. Likewise, Olavarría et al. [56] described that the macrophage activation by LPS promotes the protein kinase C activation, which in turn phosphorylates p47phox, leading to NADPH oxidase activation and reactive oxygen species generation. Thus, these cells facilitate the infection of *P. salmonis* by phagocytosis and intracellular proliferation of the bacteria. In the case of SHK-1 cells, the cytotoxicity of *P. salmonis* starts around day 5 p.i., depending on the strain and MOI used [37,57,58]. Different studies have described *P. salmonis* cytotoxicity in SHK-1 cells [37,41,58,59], indicating an apparent dependency between cytotoxicity and the number of bacteria, and more importantly, the number of live bacteria in the inoculum used. In this sense, there could be an important difference in the inoculum used in our study versus the one previously used by Levipán et al. [29], generating a higher toxicity in a shorter time. Another important factor to consider is the type of strains used for both studies. In this context, it is known that the bacterial virulence is different, especially in the in vitro systems. This was similar in the case of *P. salmonis*, which has differential characteristics in protein content, susceptibility to antibiotics, and OMV production and virulence, among others. Our results showed a delay in cell cytotoxicity caused by the bacteria, both in its planktonic and sessile form, beginning instead at 10 days p.i. Considering the *P. salmonis* cellular pathogenesis, these differences may be due to multiple factors that are intrinsic to the bacterium, related to its metabolic state, expression of virulence factors, its ability for survival and intracellular multiplication [1,59], and all this in turn may be conditioned by their planktonic and/or sessile state. In the case of *Legionella pneumophila*, its sessile state replicates in bone marrow-derived macrophage cells (BMDMs) with a greater capacity compared to its planktonic counterpart, also inducing lesser cell death in infected macrophages, similar to that observed for the EM-90 isolate used in this study. Likewise, planktonic *L. pneumophila* was rapidly sent to the lysosomes for degradation, while most biofilm-derived bacteria remained inside a vacuole [60]. This intracellular survival strategy has also been described in *P. salmonis* and could be related to a potential contribution of the biofilm in the infecting bacteria [59,61,62], revealing different degrees of cytotoxicity, a particular aspect that has not yet been studied in *P. salmonis*. Interestingly, studies carried out by Baldassarri et al. [63], revealed that *Enterococcus faecalis* strains in a sessile state showed greater survival within macrophages than mutant isogenic strains for exopolysaccharide production. Similarly, infection studies in RAW264.7 macrophages and JAWS II dendritic cells with planktonic and sessile *E. faecalis* revealed that sessile bacteria had similar or higher infection levels than their planktonic counterparts with one of the strains used showing higher survival inside macrophages than the others at 24 h, suggesting that sessile *E. faecalis* could be better adapted and overcome host defenses associated with a decreased cellular response [64]. In the case of *P. salmonis*, our results revealed marked cytotoxicity differences between the planktonic and sessile condition, and surprisingly, this cytotoxicity pattern contrasted between the LF-89 strain and the EM-90 type isolate. These findings suggest that the differences could be due to the absence or differential expression of virulence factors and a variability in secreted molecules by each bacterium during biofilm formation, consequently future studies involving these subjects may cast a new light on the differences found in *P. salmonis* LF-89 and EM-90 in its sessile and planktonic state. On the other hand, the differences in the results observed in this study compared to the results obtained by Levipán et al. [29], may be due to the use of different *P. salmonis* strains and, obviously, to the time at which the inoculum was made. Unfortunately, in both studies only one inoculum was used for experimental purposes, so it could not be compared whether this was due to time or the strains used. In light of our results, we can conclude that there would be no correlation between the cytotoxicity generated in SHK-1 cells by the sessile and planktonic condition of the same strain. However, more studies are needed to have a more global idea of the virulence of the *P. salmonis* biofilm.

Although it has been previously shown in vitro and in vivo that the virulence of planktonic *P. salmonis* is partly related to its ability to modulate the immune cell response and the host immune system [52,65], until now, no studies have compared the transcriptional response of immune genes against a planktonic and sessile *P. salmonis* infection. The NF-κB signaling pathway is an attractive target for exploitation by microbial pathogens in order to modulate host cell events, as activation of NF-κB is such a rapid response. NF-κB complexes are transferred to the nucleus within minutes after exposure to a pathogen or PAMPs. This transcription factor resides in the cytosol in an inactive form complexed to the chaperone protein inhibitory of NF-kB and IκB-α. Upon phosphorylation, IκB-α dissociates from the complex and NF-kB can enter the nucleus to bind the NF-kB responsive element in the promoter region of target genes. The participation of both NF-kB and IkB-α in the cellular responses to bacterial biofilm components have been extensively studied by transcriptional and functional analysis, being key markers to access the evasion and modulation mechanisms in the functionality of phagocytic cells. Thus, the expression of *il-1β* and *il-8* is regulated by NF-κB [66,67]. Furthermore, the immunomodulatory effect of sessile bacteria on the activity of phagocytes has been verified through the expression of cytokine encoding genes, and among them are *il-1β* and *il-8* [68,69]. Our results revealed a differential modulation on immune markers, dependent on the *P. salmonis* strain, and in some cases on the sessile/planktonic state of the bacterium (Figure 3). Overall, a significant and more attenuated response was observed for SHK-1 cells infected with sessile *P. salmonis* than to their planktonic counterpart. In the case of the LF-89 strain type, sessile bacterium generated the greatest increases in the expression of *il-1β*, *il-8*, *nf-κb*, and *ikb-α* genes compared to the planktonic form. This effect was observed only during the first hours after infection, and then returned to baseline levels after 24 h in both conditions and for all the genes analyzed. In this sense, the differential modulation of proinflammatory cytokines such as *il-1β*, *il-12*, *il-6*, and *tnf-α* were previously described in mice purified peritoneal macrophages treated with eight different isolates of sessile *P. aeruginosa* [70]. In the case of *Staphylococcus pseudintermedius*, RAW 264.7 cells macrophages treated with biofilm purified from this bacterium induced high responses of *il-1β* and *il-6*, which was attributed to the interaction of the cells with biofilm protein components, activating the signaling pathway of Toll and NF-kB receptors [71]. These results demonstrate that the interaction of the sessile form of LF-89 with SHK-1 cells was different from that obtained with the planktonic form, probably due to specific components in its exopolysaccharide matrix, which could increase the expression of the evaluated immune genes, concomitant to increased cytotoxicity of this strain in the cell line. However, bacterial sessile status is classically associated with a decreased cellular response and expression of host immune genes, which was observed in the expression levels of *il-1β*, *il-8*, and *ikb-α* (4, 6, and 12 hpi) that were significantly lower in cells infected with the sessile bacteria in EM-90 isolate. This immunosuppressive pattern was also described for *E. faecalis* in RAW264.7 cells infected with two strains of the bacterium in their sessile state, where decreases in *tnf-α* and *il-6* expression was also concomitant with increased intracellular survival of the bacteria, compared to its planktonic state [64]. Similarly, *K. pneumoniae* in biofilm mode elicited a minimal phagocytic response and cytokine expression by RAW 264.7 macrophages [72], which was confirmed in vivo by the infection of rats with sessile and dispersed bacteria from biofilm, which caused a lower innate immune response in the lungs than planktonic bacteria. Collectively, the findings of this study suggest that the increased ability of *K. pneumoniae* biofilm scattered cells to achieve surface colonization and subvert the host’s immune response grants them substantial advantages in the early stages of the infection process [73]. Likewise, some bacterial species present dysregulated proinflammatory cytokine expression during infection with sessile bacteria, which could be attributed to the modulation of the Toll-like receptor signaling pathway. Thus, *S. aureus* significantly reduced the bactericidal and proinflammatory response of RAW 264.7 cells, associated with the attenuation of *nf-κb* activation [74]. This is in accordance with *S. epidermidis*, which presented decreased production of *il-1β* and *il-6* as well as altered phagocytosis as an immune escape strategy in J774A.1 macrophages [75]. Planktonic and sessile *P. salmonis* LF-89 and EM-90 modulated the expression of *nf-κb* and its inhibitory subunit *ikb-α* in agreement with the modulation of several cytokines during the infectious process of *P. salmonis*, which has been previously described [65,76]. In this sense, the expression of immunity genes evaluated indicated increments on *il-1β*, *il-8*, and *iκb-α* genes during the 24 hpi, which later resulted in an increased cytotoxicity produced by the bacteria. Additionally, Rozas et al. [77] reported an increase in the expression of *il-1β*, *il-8*, *tnf-α*, and *ifn-ɣ* genes in the cohabiting fish kidneys at day 28 after being exposed to Trojan fish. It is important to mention that this finding was described in fish infected with both LF-89 and EM-90 strains, and the mortality in both groups began after the increment in the immune gene expression, between days 45 and 42 post challenge, respectively. Additionally, the increased *iκb-α* transcript levels in cells infected with planktonic EM-90 suggest increased activation of the *nf-kb* pathway and therefore, increased expression of proinflammatory cytokines [78,79]. Interestingly, several molecular biofilm components, specifically EPS, are important modulators of the host’s immune response against bacterial infections [69]. However, until now, the *P. salmonis* biofilm composition has not been described, nor are there any studies evaluating the effect of biofilm production by *P. salmonis* during in vitro or in vivo infection, nor its association with the immune response. According to our results, immune genes are differentially modulated in a manner that is directly proportional between the cytotoxicity of the planktonic or sessile bacteria and the increments in the expression of *il-1β*, *il-8*, and *iκb-α* genes. The *P. salmonis* sessile and planktonic conditions modulated bacterial virulence in SHK-1 cells. Levipán et al. (2020) [29] observed that *P. salmonis* forms viable, stable, and fish skin-mucus tolerant biofilms on abiotic surfaces in aquaculture. Thus, the biofilm produced by *P. salmonis* could be considered an important virulence factor as well as a good candidate for the future development of new and better antimicrobial agents. In this sense, the anti-biofilm activity is based on inhibiting bacterial adhesion to surfaces, the disruption of the biofilm architecture during its maturation process, and the interference with the quorum sensing system [80]. Additionally, gene expression regulation by planktonic and sessile *P. salmonis* could be considered a key component that affects the immune response of fish. The study of the antigenic components in biofilm opens up new possibilities for the development and application of biofilm-based vaccine prototypes as well as a prophylactic strategy against bacterial diseases, which has already been explored in tilapia and carp with promising results [81,82].

## 5. Conclusions

This study determined the biofilm production levels of four strains belonging to both *P. salmonis* genogroups. Additionally, we determined the differential virulence of these strains in their two planktonic and sessile stages in SHK-1 cells as well as the immune gene expression induced by these two bacterial conditions. In conclusion, these results demonstrate that the planktonic or sessile condition of *P. salmonis* generated a differential pattern of virulence and gene expression and this is, apparently, dependent on each strain and bacterial condition evaluated. While the specific role of biofilm in virulence was not clearly elucidated in this study, the presence and abundance of biofilm suggest an important biological function by interacting with host proteins and modulating its immune response, and thus affecting bacterial pathogenesis. However, further studies are needed to clarify the presence of *P. salmonis* biofilm during infection, and also to establish the specific role of the components of this biofilm, especially regarding possible functions inside the host cells.

## Figures and Tables

**Table 1:** Primers used for gene expression analysis.

Gene	Sequence (5′- > 3′)	GenBank Accession No.
*ef-1* *α*	F-CCCCTCCAGGACGTTTACAAA R-CACACGGCCCACAGGTACA	NM_001123629.1
*il-1* *β*	F-CAAGCTGCCTCAGGGTCT R-CGGCACCCTTTAACCTCTCC	NM_001123582.1
*il-8*	F-GCAACAGCGGTCAGGAGATT R-TGGAATGATTCCCCTTCTTCA	HM162835.1
*nf-* *κ* *b*	F-ACCTGGCCATCATTCACCAG R-TGGTTGAGCTTGTCGAGGAC	NM_001173583.1
*Iκb-α*	F-GGAGAGTGAGGAGGAGTGCAT R-CTGCTTCAATTCTGCCCAAATGTAA	XM_014204687.1

## Supplementary Materials

The following are available online at https://www.mdpi.com/2076-2607/8/10/1609/s1, Figure S1: Cell viability of planktonic and sessile *P. salmonis.*
