# Supplementary figures and images for: Biofilm Produced In Vitro by Piscirickettsia salmonis Generates Differential Cytotoxicity Levels and Expression Patterns of Immune Genes in the Atlantic Salmon Cell Line SHK-1
Source: Microorganisms. 2020 Oct 20;8(10):1609. doi: 10.3390/microorganisms8101609 (PMC7594049; doi:10.3390/microorganisms8101609)

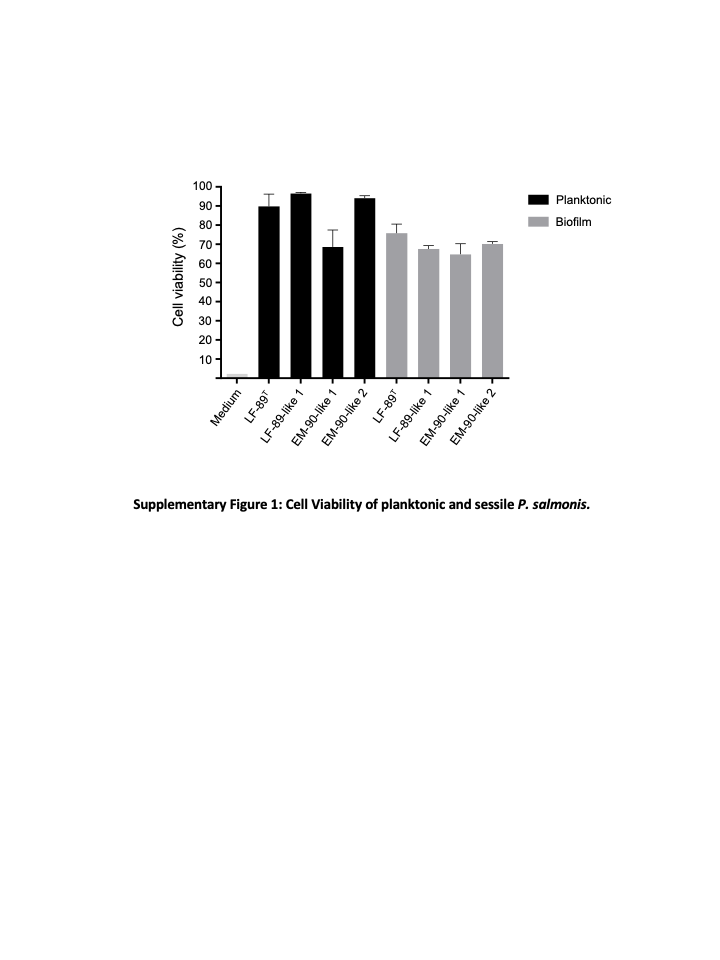

Supplement: Supplementary file 1 [file microorganisms-08-01609-s001.zip › microorganisms-910192-supplementary.tiff]
